# Mini-invasive surgical instruments in transaortic myectomy for hypertrophic obstructive cardiomyopathy: a single-center experience with 168 cases

**DOI:** 10.1186/s13019-021-01403-3

**Published:** 2021-03-17

**Authors:** Qiang Ji, Yu Lin Wang, Ye Yang, Hao Lai, Wen Jun Ding, Li Min Xia, Chun Sheng Wang

**Affiliations:** 1 Department of Cardiovascular Surgery of Zhongshan Hospital Fudan University, 180 Fenglin Rd, Shanghai, 200032 China; 2 Shanghai Municipal Institute for Cardiovascular Diseases, 1609 Xietu Road, Shanghai, 200032 China; 3 Department of Cardiovascular Surgery of Xiamen Branch of Zhongshan Hospital Fudan University, 668 Jinhu Road, Huli District, Xiamen, 510530 China

**Keywords:** Septal myectomy, Hypertrophic obstructive cardiomyopathy, Mini-invasive surgical instruments, Left ventricular outflow tract gradient, Mitral regurgitation

## Abstract

**Background:**

Although septal myectomy is a standard strategy for managing patients with hypertrophic obstructive cardiomyopathy (HOCM) and drug-refractory symptoms, so far, only a few experienced myectomy centers exist globally. Mainly, this can be explained by the many technical challenges presented by myectomy. From our clinical experience, applying the mini-invasive surgical instruments during myectomy potentially reduces the technical difficulty. This study reports the preliminary experience regarding transaortic septal myectomy using mini-invasive surgical instruments for managing patients with HOCM and drug-refractory symptoms; also, we evaluate the early results following myectomy.

**Methods:**

Between March 2016 and March 2019, consecutive HOCM patients who underwent isolated transaortic septal myectomy using the mini-invasive surgical instruments were enrolled in this analysis. Intraoperative, in-hospital and follow-up results were analyzed.

**Results:**

We included 168 eligible patients (83 males, mean 56.8 ± 12.3 years). The midventricular obstruction was recorded in 7 (4.2%) patients. All patients underwent transaortic septal myectomy with a mean aortic cross-clamping time of 36.0 ± 8.1 min. During myectomy, 9 (5.4%) patients received repeat aortic cross-clamping. Surgical mortality was 0.6%. Notably, 5 (3.0%) patients developed complete atrioventricular block, they needed permanent pacemaker implantation. The median follow-up time was 6 months; however, no follow-up deaths occurred with a significant improvement in New York Heart Association functional status. We reported a sharp decrease in the maximum gradients from the preoperative value (11.6 ± 7.4 mmHg vs. 94.4 ± 22.6 mmHg, *p* < 0.001). The median degree of mitral regurgitation fell to 1.0 (vs. 3.0 preoperatively, *p* < 0.001) with a significant reduction in the proportion of moderate or more regurgitation (1.2% vs. 57.7%, *p* < 0.001).

**Conclusions:**

Mini-invasive surgical instruments may be beneficial in reducing the technical challenges of transaortic septal myectomy procedure. Of note, transaortic septal myectomy using the mini-invasive surgical instruments may present with favorable results.

## Introduction

Septal myectomy, which effectively relieves left ventricular outflow tract (LVOT) obstruction, reliably reduces mitral regurgitation (MR), and thus substantially relieves symptoms and greatly improves the quality of life, has been adopted as a standard treatment option for patients with hypertrophic obstructive cardiomyopathy (HOCM) and drug-refractory symptoms [1–6]. However, only a few experienced myectomy centers are available globally; this is mainly because this technique requires a relevant operator and institutional experience, posing challenges in developing standard surgical approaches for HOCM management [7–11]. Also, septal myectomy requires appropriate depth and adequate length of septal excision through limited surgical exposure, which may predispose patients to severe complications, including iatrogenic free wall rupture, septal perforation, complete atrioventricular block, or recurrent obstruction [12, 13]. Owing to the high technical challenges posed by myectomy, numerous studies have been geared towards exploring reliable approaches for improving myectomy.

The high technical difficulty of myectomy is mainly posed by the appropriate depth and adequate length of septal excision under unfavorable exposure conditions. Other reports show that an adequate length of septal excision is extremely important to surgical effect [14]. During the past several years, mini-invasive surgical instruments including long-handled forceps and scissors have been employed for septal myectomy in our center. From our clinical experience, mini-invasive surgical instruments during myectomy may ensure adequate resection length. Also, the small operational radius is potentially beneficial as this reduces challenges posed by myectomy under difficult exposure conditions. Herein, we report the preliminary experience regarding transaortic septal myectomy using mini-invasive surgical instruments for managing patients with HOCM and drug-refractory symptoms and evaluate the early results.

## Methods

### Patients

We reviewed data for consecutive HOCM patients aged ≥18 years who underwent septal myectomy using the mini-invasive surgical instruments in our center between March 2016 and March 2019. Inclusion criteria included: (1) the maximum LVOT gradients ≥50 mmHg at rest or with provocation; and (2) presence of severe symptoms refractory to maximum pharmacologic therapy with non-vasodilating β-blockers and/or calcium channel blockers. Exclusion criteria included: (1) organic mitral valve (MV) lesions (rheumatic, degenerative, ischemic, infective, and mitral annulus calcification); (2) previous valvular surgery; (3) LVOT obstruction secondary to hypertensive heart disease or severe aortic stenosis; (4) concomitant coronary artery disease requiring bypass grafting; (5) concomitant modified Maze procedure; (6) concomitant obstruction of right ventricular outflow tract requiring enlargement; and (7) patients who underwent septal myectomy via the transseptal approach through right atrium or via the left atrial approach through interatrial sulcus.

### Preoperative evaluation

Preoperative transthoracic echocardiography (TTE) examination aided in defining: (1) the location and magnitude of left ventricular pressure gradient, both at rest and with provocation; (2) the distribution and severity of myocardial hypertrophy; (3) MV anatomy and function; (4) mitral subvalvular anomalies, including abnormal chordae tendineae attached to the ventricular septum or free wall (false cords), fibrotic and retracted secondary chordae inserted on the anterior mitral leaflet body, and papillary muscle (PM) abnormalities (hypertrophy, and direct insertion into the anterior mitral valve leaflet); and (5) intrinsic MV diseases, including lesions of mitral leaflets and mitral annulus. Resting LVOT velocity was evaluated via continuous-wave Doppler of the outflow tract from an apical window. An estimate of the resting LVOT pressure gradient was established using the modified *Bernoulli* equation (i.e., gradient = 4v^2^, where v = peak LVOT velocity). For symptomatic patients with resting LVOT gradients < 30 mmHg, the Valsalva maneuver and stand-to-squat were frequently employed. Furthermore, we relied on cardiac magnetic resonance frequently to measure basal septal thickness and characterize PM morphology and location within the left ventricular cavity, PM thickness, and PM mobility.

### Study protocol

This study protocol was approved by the ethics committee of *Zhongshan Hospital Fudan University* and was consistent with the *Declaration of Helsinki*. All enrolled patients signed an informed consent approved by the ethics committee. Two trained staff actively collected all data; however, they were not informed of the purpose of this study so as not to bias the results.

Using a standard data collection form, the baseline and surgical characteristics, intraoperative and in-hospital results were collected retrospectively from our institutional database, then reviewed. Intraoperative results included: the incidence of repeat aortic cross-clamping (due to inadequate septal myectomy, iatrogenic free wall rupture, iatrogenic septal perforation, and iatrogenic aortic valve perforation) and transesophageal echocardiography data, including the maximum LVOT gradient, interventricular septal thickness, systolic anterior motion (SAM), the severity of MR, and aortic regurgitation. In-hospital results included: surgical death, complete atrioventricular block requiring a permanent pacemaker, new-onset atrial fibrillation, complete left bundle branch block, new-onset cerebrovascular adverse events, prolonged mechanical ventilation (> 72 h), redo for bleeding, and postoperative hospital stay. Surgical death was defined as all deaths within 30 days of operation regardless where death occurred and all in-hospital deaths after 30 days among patients who had not been discharged after the initial operation.

Patients were regularly followed up at 3 and 6 months following myectomy and in 6-month intervals thereafter. Follow-up data were collected prospectively through clinic visits or telephone interviews, including results on the survival, reoperation for recurrent LVOT obstruction and/or symptomatic MR, New York Heart Association (NYHA) functional class at the latest follow-up. Also, echocardiographic data at the latest follow-up including residual obstruction (the maximum gradient ≥30 mmHg), septal thickness, SAM, residual MR, and ventricular septal defect were also recorded. In addition, the incidence of major adverse events (including deaths, complete atrioventricular block, residual obstruction, and residual moderate or more MR) per surgeon was calculated and compared.

### Surgical procedures

**Fig. 1:**
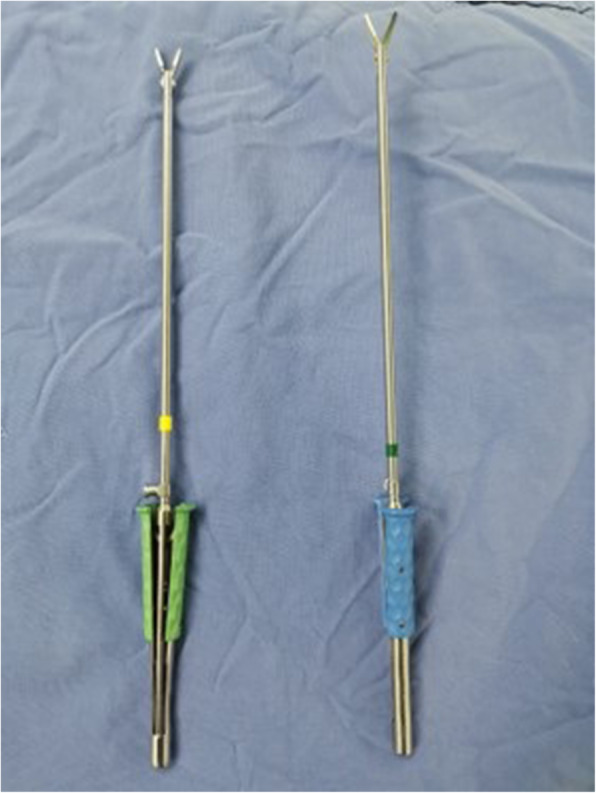
Mini-invasive surgical instruments. Special surgical instruments include long surgical forceps (the left) and long arm surgical scissors (the right)

Operations were guided by intraoperative transesophageal echocardiography (TEE). Particular attention was paid to ventricular septal anatomy as well as thickness, MV anatomy and function, and mitral subvalvular anatomy. Each patient was put in the reverse *Trendelenburg* and left lateral decubitus position. Under general anaesthesia, the heart and ascending aorta were exposed via a median incision with sternotomy. Cardiopulmonary bypass with ascending aortic and right atrial cannulation was established with a left ventricular vent placing via the right superior pulmonary vein. Employing a low oblique aortotomy (approximately 7–10 mm above the right coronary ostium), the aortic valve leaflets were pulled up to permit access to the outflow tract, the hypertrophic cardiac muscle, anterior MV leaflet, and mitral subvalvular apparatus. We then used a headlamp and loupe magnification to achieve a better inspection of the left ventricular cavity. The mini-invasive surgical instruments (see Fig. 1) were applied in the resection process. Scalpel resection was usually initiated at the nadir of the right cusp, 5 mm below the aortic valve, extending leftwards to the left trigone. The septal excision area was lengthened beyond the bases of PMs and toward the heart apex. The resection depth was up to 50% of the basal septum thickness. The excision of the hypertrophic muscles as a whole mass was necessary. Moreover, mitral subvalvular anomalies were corrected, including false cords cutting, retracted secondary chordae cutting, and hypertrophic PM release and/or resection. After completing the resections, the bases of the PMs were visible through the incision of the aortic root. The outflow tract, mitral and aortic valves were precisely and extensive re-explored.

We employed TEE after weaning off bypass to evaluate the maximum gradients and the severity of MR following myectomy. Immediately, repeat aortic cross-clamping was performed if there was residual obstruction and/or residual moderate or more MR or a ventricular septal defect or a left ventricular free wall rupture.

### Statistical analysis

All statistical data were analyzed with the SPSS statistical package version 22.0 (SPSS Inc., Chicago, IL, USA). Categorical data were expressed as frequency distributions; comparison of single percentages was achieved between groups using *Fisher’s* exact test if the expected frequency was < 5 or the *chi-square* test. Normally distributed continuous variables were expressed as the mean ± standard deviation; here, comparisons between groups were established using an independent-samples *t*-test. Non-normally distributed continuous variables were expressed as the median and interquartile range (IQR); here comparison between groups was achieved using the *Wilcoxon* rank sum test. A two-sided *p*-value less than 0.05 was considered statistically significant.

## Results

### Study population

We did a review of 208 adult patients who met the inclusion criteria. Later, 40 patients were excluded due to: concomitant valvular heart disease requiring surgery (27 patients), concomitant Maze procedure (3 patients), concomitant enlargement of right ventricular outflow tract (2 patients), and those who underwent septal myectomy via a transseptal approach through right atrium (8 patients). Eventually, 168 eligible patients (83 male patients and 85 female patients with a mean age of 56.8 ± 12.3 years) were enrolled for data analysis. We have listed the baseline characteristics in Table 1. Although 39.9% of the population had a history of hypertension, it was not deemed severe enough to be the primary cause of ventricular hypertrophy. Notably, 9 (5.4%) patients underwent previous alcohol septal ablation. Atrial fibrillation and right bundle branch block were recorded in 14 (8.3%) patients and 4 (2.4%) patients, respectively. All patients manifested severe limiting symptoms, including dyspnea, angina-like chest pain, and syncope, with New York Heart Association (NYHA) functional class III and IV in 86.3% of the population.

**Table 1:** Baseline and surgical characteristics

Variables	Value
**Demographics**	
Age (years)	56.8 ± 12.3
Gender (Females/Males)	85/83
Obesity (Body mass index > 30 kg/m^2^)	9 (5.4%)
**Concomitant diseases**	
Diabetes mellitus	16 (9.5%)
Coronary artery disease	7 (4.2%)
Hypertension	67 (39.9%)
Chronic obstructive pulmonary disease	13 (7.7%)
Cerebrovascular disease	9 (5.4%)
Family history of HCM	16 (9.5%)
Family history of sudden death	6 (3.6%)
**Preoperative cardiac status**	
NYHA class	
II	23 (13.7%)
III	120 (71.4%)
IV	25 (14.9%)
Previous alcohol septal ablation	9 (5.4%)
AF	14 (8.3%)
Left BBB	3 (1.8%)
Right BBB	4 (2.4%)
**Preoperative TTE data**	
Maximum LVOT gradient (mmHg)	94.4 ± 22.6
Interventricular septal thickness (mm)	18.3 ± 3.1
SAM	168 (100%)
Degree of MR (median, IQR)	3.0 (2.0–3.0)
Moderate or more MR	97 (57.7%)
Midventricular obstruction	7 (4.2%)
LVEF (%)	67.0 ± 4.2
LVEDD (mm)	44.6 ± 4.5
Mitral subvalvular anomalies	45 (26.8%)
False cords	11 (6.5%)
Retracted secondary chordae	29 (17.3%)
PM abnormalities	13 (7.7%)
**Surgical characteristics**	
ACC time (min)	36.0 ± 8.1
Transaortic myectomy alone	123 (73.2%)
Myectomy plus sub-MV management	45 (26.8%)
False cords cutting	11 (6.5%)
Retracted secondary chordae cutting	29 (17.3%)
PM release and/or resection	13 (7.7%)

*HCM* hypertrophic cardiomyopathy, *NYHA* New York Heart Association, *AF* atrial fibrillation, *BBB* bundle branch block, *TTE* transthoracic echocardiography, *LVOT* left ventricular outflow tract, *SAM* systolic anterior motion, *IQR* interquartile range, *MR* mitral regurgitation, *LVEF* left ventricular ejection fraction, *LVEDD* left ventricular endo-diastolic diameter, *PM* papillary muscle, *ACC* aortic cross-clamping, *MV* mitral valve

Upon TTE examination, a mean maximum LVOT gradient of 94.4 ± 22.6 mmHg with a mean interventricular septal thickness of 18.3 ± 3.1 mm was reported. SAM was reported in all patients, of whom 97 (57.7%) were diagnosed with moderate or more MR. The midventricular obstruction was recorded in 7 (4.2%) patients. Mitral subvalvular anomalies were recorded in 45 (26.8%) patients, including false cords (11 patients), fibrotic and retracted secondary chordae (29 patients), and PM abnormalities (13 patients).

All patients underwent transaortic septal myectomy with a mean aortic cross-clamping time of 36.0 ± 8.1 min (median, 35.0 min). A total of 45 (26.8%) patients received mitral subvalvular procedures in addition to myectomy, including false cords cutting (11 patients), fibrotic and retracted secondary chordae cutting (29 patients), and PM release and/or resection (13 patients).

### Intraoperative results

Here, we recorded repeat aortic cross-clamping in 9 (5.4%) patients; 5 of them presented with a residual obstruction or moderate or more MR due to inadequate initial septal myectomy. Among the 5 patients, 4 underwent a “more” extended myectomy in terms of depth and length and/or leftward direction from the left ventricular free wall toward the MV according to TEE findings, whereas one patient received transaortic mitral valve repair using the “edge-to-edge” technique. Another 2 patients underwent repair of a left ventricular free wall rupture due to free wall rupture. The remaining 2 patients underwent repairs of the septal defect and aortic right valve due to iatrogenic perforation, respectively.

Upon TEE examination, the maximum LVOT gradients following myectomy fell to 10.8 ± 6.3 mmHg with 13.8 ± 2.1 mm as the interventricular septal thickness, both of which were significantly lower than the preoperative values (*p* < 0.001). No residual obstruction was recorded. SAM was reported in 16 (9.5%) patients, of whom one showed moderate MR. However, we reported no instances of more than moderate MR.

### In-hospital outcomes

One patient died of cerebral hernia on the fourth day postoperatively, which may be associated with acute cerebral infarction as revealed by skull computed tomography examination. Surgical mortality was 0.6%. Five (3.0%) patients developed complete atrioventricular node block and required permanent pacemaker implantation. Of note, among the 5 patients, 3 had right bundle branch block prior to surgery. Another 4 (2.4%) patients developed new-onset atrial fibrillation, but all returned to sinus rhythm following electrical cardioversion. Other postoperative complications are listed in Table 2. A total of 167 patients were discharged, and the median length of postoperative hospital stay was 6 days.

**Table 2:** Clinical outcomes and echocardiographic results

Variables	Value
**Intraoperative**	
Repeat aortic cross-clamping	9 (5.4%)
Inadequate septal myectomy	5 (3.0%)
Iatrogenic free wall rupture	2 (1.2%)
Iatrogenic septal perforation	1 (0.6%)
Iatrogenic AV perforation	1 (0.6%)
TEE data	
Maximum LVOT gradient (mmHg)	10.8 ± 6.3
Interventricular septal thickness (mm)	13.8 ± 2.1
SAM	16 (9.5%)
MR severity (median, IQR)	1.0 (1.0–1.0)
Moderate MR	1 (0.6%)
More than moderate MR	0
Moderate or more aortic regurgitation	0
**In-hospital**	
Surgical death	1 (0.6%)
CAVB	5 (3.0%)
Right BBB prior to surgery	3
New-onset AF	4 (2.4%)
Complete left BBB	69 (41.1%)
Cerebrovascular adverse events	2 (1.2%)
Prolonged ventilation (> 72 h)	3 (1.8%)
Redo for bleeding	1 (0.6%)
Postoperative hospital stay (median, IQR; days)	6 (5–6)
**Follow-up**	
Number of patients	161 (95.8%)
Duration of follow-up (median, IQR; months)	6.0 (6.0–13.0)
Survival	161 (100%)
Reoperation	0
NYHA class at the latest follow-up	
I	127 (78.9%)
II	34 (21.1%)
TTE data at the latest follow-up	
Maximum LVOT gradient (mmHg)	11.5 ± 7.4
Residual obstruction	3 (1.9%)
Septal thickness (mm)	14.0 ± 2.5
SAM	9 (5.6%)
Moderate MR	2 (1.2%)
Ventricular septal defect	1 (0.6%)

*AV* aortic valve, *IQR* interquartile range, *TEE* transesophageal echocardiography, *CAVB* complete atrioventricular block

### Follow-up results

We made a follow-up visit to 161 (95.8%) patients with a median duration of 6 (IQR, 6–13) months. During the follow-up periods, the clinical manifestations disappeared, and no deaths or re-interventions were observed. NYHA functional class significantly improved from the preoperative values, and no patients was classified in class III or IV.

TTE examination at the latest follow-up showed the maximum LVOT gradients were significantly lower than the preoperative values (Table 2). Three patients developed residual obstruction with the maximum gradients of 34 mmHg, 39 mmHg, and 42 mmHg, respectively; however, they did not receive repeat surgery or alcohol septal ablation. Moderate MR at the latest follow-up was recorded in 2 (1.2%) asymptomatic patients (Table 2), which was significantly lower than the preoperative value (1.2% vs. 57.7%, *p* < 0.001). No cases of severe MR at follow-up were recorded. Of note, one patient who developed iatrogenic septal perforation intraoperatively presented with one 2-mm asymptomatic ventricular septal defect during follow-up. We classified this patient as NYHA class I and proceeded with dynamic evaluation.

### Incidence of major adverse events per surgeon

In our center, mini-invasive surgical instruments have been used routinely for transaortic myectomy. Six surgeons (total number of myectomy procedures during the three-year period of 72, 36, 23, 14, 13, and 10, respectively) have completed 168 transaortic myectomy procedures using mini-invasive surgical instruments. A total of 11 major adverse events were recorded, including surgical death (one patient), complete atrioventricular block (5), residual obstruction (3), and moderate residual MR (2). As shown in Table 3, no significant difference was found among all surgeons regarding the incidence of major adverse events (*p* = 0.739).

**Table 3:** Incidence of major adverse events per surgeon

Number	^a^Volume	^b^Major adverse events (number of patients)	Incidence	p
Surgeon 1	72	CAVB (2), residual MR (1)	4.2%	0.739
Surgeon 2	36	Death (1), CAVB (1), residual obstruction (1)	8.3%	
Surgeon 3	23	CAVB (1), residual obstruction (1)	8.7%	
Surgeon 4	14	CAVB (1)	7.1%	
Surgeon 5	13	Residual MR (1)	7.7%	
Surgeon 6	10	Residual obstruction (1)	10.0%	

^a^Volume, total number of myectomy procedures during the three-year period

^b^Major adverse events, postoperative adverse events including deaths, complete atrioventricular block, residual obstruction, and residual moderate or more mitral regurgitation

## Discussion

In the 1960s, Morrow et al. reported transaortic resection of a small amount of muscle from the proximal ventricular septum, a technique generally described as classic Morrow operation. Over the next decades, myectomy has evolved from the classic Morrow operation to a more extended septal myectomy guided by preoperative cardiovascular magnetic resonance and intraoperative TEE [15–18]. The high technical difficulty, however, limited clinical application of the extended septal myectomy procedure. Later, Ferrazzi et al. in 2015 proposed the transaortic secondary chordae cutting in addition to a shallow septal myectomy for managing HOCM [8]; notably, it was associated with favorable results [8, 19]. However, such an approach remained controversial because the secondary chordae maintained ventricular geometry and enhanced wall thickening, which may be helpful in case of left ventricular dilation and systolic dysfunction [20]. In this study, using the mini-invasive surgical instruments, transaortic septal myectomy was performed in 168 patients with HOCM and drug-refractory symptoms. Favorable results were achieved, including a low surgical mortality (< 1%) with no deaths or re-interventions at follow-up, a significant improvement of life quality with recovery from symptoms and NYHA functional class I and II, effective relief of LVOT obstruction, and a reliable MR reduction. The observed good survival and a significant improvement in NYHA functional status coincided with an effective relief of LVOT obstruction and a reliable reduction of MR, which validated the safety and efficacy of transaortic septal myectomy using the mini-invasive surgical instruments for treating symptomatic HOCM patients.

In this series, including 168 transaortic myectomy procedures with the aid of the mini-invasive surgical instruments, the mean duration of aortic cross-clamping was 36.0 ± 8.1 min (median, 35.0 min), significantly lower than the value (median, 68 min) reported from a famous and experienced myectomy center in the same country [21]. The shorter aortic cross-clamping time in this series may be explained by the application of the mini-invasive surgical instruments, regarding that the small operational radius of the mini-invasive surgical instruments is potentially beneficial to reduce the challenges posed by myectomy under difficult exposure conditions.

Note that 7 HOCM patients who suffered from midventricular obstruction were subjected to transaortic septal myectomy using the mini-invasive surgical instruments. Having demonstrated favorable results, the application of the mini-invasive surgical instruments made it easy for myocardium removal at the base of PMs, or extend it to the apex of the left ventricle. For surgeons, surgical comfort is essential, especially for those without experience when performing septal myectomy procedures. These may attribute to the small operational radius of the mini-invasive surgical instruments, highly crucial in case of limited surgical exposure. Moreover, the application of the mini-invasive surgical instruments is beneficial for an adequate length of septal excision, which is extremely important to surgical effect [14].

Six surgeons with greatly varied myectomy-volume have completed 168 transaortic myectomy procedures using mini-invasive surgical instruments. Favorable results were achieved, and no significant difference was observed among six surgeons regarding the incidence of major adverse events, which suggested that transaortic myectomy using mini-invasive surgical instruments for managing HOCM patients may receive good and reproducible results.

Major complications, including complete atrioventricular block, left ventricular free wall rupture, aortic valve injury and iatrogenic septal perforation were associated with septal myectomy. In the present cohort, the incidence of pacemaker implantation (3.0%) was greater than expected based on other large series [22, 23]. Of note, this elevated incidence was attributed to the excessive subaortic resection to the side of the noncoronary valve following incision made just to the right of the nadir of the right aortic sinus. More than 41.0% of patients developed a complete left bundle branch block after myectomy, which permitted the adoption of conservative surgical strategies in patients with preoperative right bundle branch block. In this series, left ventricular free wall rupture, a rare complication after myectomy, occurred in 2 (1.2%) patients. We successfully managed the complication via cardiopulmonary bypass and cardioplegic arrest with a double-armed 3–0 polypropylene suture with a pledget placed in a horizontal mattress fashion, similar to the technique described to control a stab wound to the heart in close proximity myectomy to a coronary artery. Although we corrected abnormal papillary muscles, left ventricular free wall rupture was not associated with the excision of muscle bundles based on the operative exploration. Besides, left ventricular free wall rupture may be associated with excessive subaortic resection to the mitral anterior commissure. Aortic valve injury and iatrogenic septal perforation were common in young patients with small aortic roots and old patients without severe septal hypertrophy, respectively. In our series, ventricular septal perforation occurred in one female patient aged 70 years with a 17.0 mm preoperative septal thickness. Although the perforation was repaired using a bovine pericardium patch, a residual ventricular septal defect was evident during follow-up and could be associated with the thin myocardium surrounding the perforation and with the small patch that was not large enough to extend beyond the edge of the perforation at a certain distance.

This study had some potential limitations. First, this being a single-center, single-armed observational study with limited sample size, may have influenced the generalizability of the results. A control group including patients undergoing myectomy without the aid of the mini-invasive surgical instruments was not established, and the number of patients is relatively small, implying potential weakness of the results. Second, only a minor part of patients received cardiac magnetic resonance imaging, as it did not serve as a regular examination modality in the early times at our institution. Third, a functional capacity assessment tool, such as the 6-min walking test, and a quality-of-life assessment tool, such as the SF-36 questionnaire, were not employed. Finally, the duration of follow-up was relatively short, longer observations would be critical to confirm our findings.

## Conclusion

The present study demonstrated that transaortic septal myectomy procedures employing mini-invasive surgical instruments may be beneficial to reduce the underlying technical challenges. Furthermore, the transaortic septal myectomy approach using the mini-invasive surgical instruments may present with favorable results.

## Data Availability

The datasets used in the current study are available from the corresponding author or the first author on reasonable request.

## Abbreviations

LVOT: Left ventricular outflow tract
MR: Mitral regurgitation
HOCM: Hypertrophic obstructive cardiomyopathy
MV: Mitral valve
TTE: Transthoracic echocardiography
PM: Papillary muscle
NYHA: New York Heart Association
SAM: Systolic anterior motion
TEE: Transesophageal echocardiography
